# Perceptions and attitudes towards AI among trainee and qualified radiologists at selected South African training hospitals

**DOI:** 10.4102/sajr.v29i1.3026

**Published:** 2025-01-10

**Authors:** Ayanda I. Nciki, Linda T. Hlabangana

**Affiliations:** 1 Department of Radiology, Faculty of Health Sciences, University of the Witwatersrand, Johannesburg, South Africa

**Keywords:** artificial intelligence, attitudes, perceptions, qualified radiologist, trainee radiologist

## Abstract

**Background:**

Artificial intelligence (AI) is transforming industries, but its adoption in healthcare, especially radiology, remains contentious.

**Objectives:**

This study evaluated the perceptions and attitudes of trainee and qualified radiologists towards the adoption of AI in practice.

**Method:**

A cross-sectional survey using a paper-based questionnaire was completed by trainee and qualified radiologists. Survey questions covered AI knowledge, perceptions, attitudes, and AI training in the registrar programme on a 3-point Likert scale.

**Results:**

A total of 100 participants completed the survey; 54% were aged 26–65 years and 61% were female, with none currently using AI in daily radiology practice. The majority (78%) of participants understood the basics and knew the role of AI in radiology. Most knew about AI from media reports (77%) and majority (95%) were never involved in AI training; only 3% of participants had no knowledge of AI at all. Participants agreed that AI could reliably detect pathological conditions (89%), reach reliable diagnosis (89%), improve daily work (78%), and 89% favoured AI practice; 89% believed that in the future, machine learning will not be independent of the radiologist. Participants were willing to learn (98%) and contribute towards advancing AI software (97%) and agreed that AI will improve the registrars’ programme (97%), also noting that AI applications are as important as medical skills (87%).

**Conclusion:**

The findings suggest AI in radiology is in its infancy, with a need for educational programmes to upskill radiologists.

**Contribution:**

Participants were positive about AI implementation in practice and in the registrar learning programme.

## Introduction

Artificial intelligence (AI) is an advancing field of computer science that builds machines that utilise data to perform cognitive tasks that require human intelligence.^1,2,3^ Currently, the development and availability of highly sophisticated machines, pattern recognition algorithms and software that harness computational power to perform complex tasks in medical imaging and bioinformatics is ever-increasing.^1^ The adoption of AI in medicine and radiology has the potential to revolutionise the field while optimising patient care.^1^ Utilisation of AI will most likely increase accuracy of diagnosis, prognosis and treatment of diseases while enhancing efficiency and reducing the workload of medical practitioners.^2,4^

In radiology, digital images have been implemented since the beginning of the 21st century, making the field well-positioned to deploy AI technologies as there now exists a large repository of data ready to be translated.^4^ Ideally, for patient images generated using various imaging modalities such as CT, mammogram, MRI, ultrasound and X-ray, AI technology can be deployed to determine areas of interest and diagnosis.^4^ Despite AI having been adopted to perform specific tasks in the field of radiology, much to the delight and embrace of many radiologists,^5,6^ some radiologists remain apprehensive. Several factors, ranging from lack of adequate information, demographics, religion and mistrust, influence people’s reaction to AI technologies,^7^ while others simply fear job and turf losses (shift of practice or takeover of radiological examinations by other disciplines).^8,9^ In addition, there are fears concerning the ethical and safety concerns surrounding AI in medicine with some radiologists predicting that patients would not accept the technology.^3,5,7,10,11^

Although studies exist on perceptions and attitudes of healthcare practitioners, including radiologist and radiology students, on AI technologies,^5,6,8,9,10^ there is a noticeable research gap regarding perceptions and attitudes of trainee and qualified radiologist in the African context. Studies in Africa are limited to Nigeria^12^ and Kenya^13^ and showed that knowledge of AI in radiology was limited; however, both trainee and qualified radiologists had positive attitudes if AI was to be fully integrated into daily practice. Most participants in these studies believed AI would play a pivotal role in the future practice of radiology and acceptability of the technology was dependent on the level of knowledge of their applications in medical imaging.

South Africa, like many other African countries, grapples with inadequate human resources, unequal distribution of health professionals, including specialists such as radiologists, between private and public sectors, and provinces, as well as resource shortages which might affect the implementation of AI.^14^ Adoption of AI in radiology could ease the burden on the limited crop of specialist radiologists and augment their shortage. However, implementation of this technology would require buy-in from current practicing radiologists, thus it is important to understand their current state of knowledge and perceptions towards it. On the other hand, the opinions of trainee radiologists are particularly important as they are the next generation of radiologists who are expected to be impacted the most by the advent and advancement of AI technologies in the field.

The aim of this study was to evaluate the perceptions and attitudes of qualified and trainee radiologists, from six training hospitals in University of the Witwatersrand radiology training circuit in South Africa, towards the adoption of AI in clinical practice.

## Research methods and design

### Study design and participants

The cross-sectional survey consisted of 25 questions divided into four sections, each designed to ask questions related to participants’ demographic information and to rate their knowledge, perceptions and attitudes towards AI, respectively, using a 3-point Likert scale. The questionnaire was administered to trainee (registrars), and qualified radiologists based at six academic hospitals within the University of Witwatersrand radiology circuit, namely, Charlotte Maxeke Johannesburg Academic Hospital, Chris Hani Baragwanath Academic Hospital, Rahima Moosa Mother and Child Hospital, Helen Joseph Hospital, Thelle Mogoerane Regional Hospital and Klerksdorp Tshepong Hospital. Trainee radiologists were defined as medical practitioners with a Bachelor of Medicine degree training to specialise in radiology and qualified radiologists were those with radiology-specific certifications regardless of their level of experience post-qualification. Participation was voluntary and was not associated with any duties and/or activities of the trainees nor qualified radiologists at the time of the research.

### Sampling design

Minimum sample size required for this cross-sectional study was determined using Equation 1:

$$N=\left[Z^{2}P(1-P)/D^{2}\right]$$ [Eqn 1]

assuming a prevalence rate of 9% against the use of AI in radiology (*P*) at a 95% confidence interval (*D* = 0.05), and normal standard deviation at 80% power of 1.96 (*Z*), the minimum sample size was determined as N = 132. To avoid collecting unnecessary data, the purposive sampling technique was used to recruit participants more likely to offer relevant information for answering the research question.^12^

### Data analysis

Participants’ responses from the questionnaire were recorded and processed using Microsoft Excel (Microsoft, RedMond, WA, United States). A de-identified Excel spreadsheet was imported into Stata Version 18 (StataCorp, College Station, TX, United States) for further analysis. Categorical variables were presented as frequencies and percentages.

### Ethical considerations

Ethics approval was obtained from the Human Research and Ethics Committee of the University of the Witwatersrand (Approval Number: MED230910). Further permission to conduct the research with the registrars and radiologists affiliated to the University of the Witwatersrand within academic hospitals was obtained from the Academic Head of Radiology in the University of the Witwatersrand. Prior to each participant filling out the survey, their informed consent was acquired.

## Results

### Demographics

**FIGURE 1:**
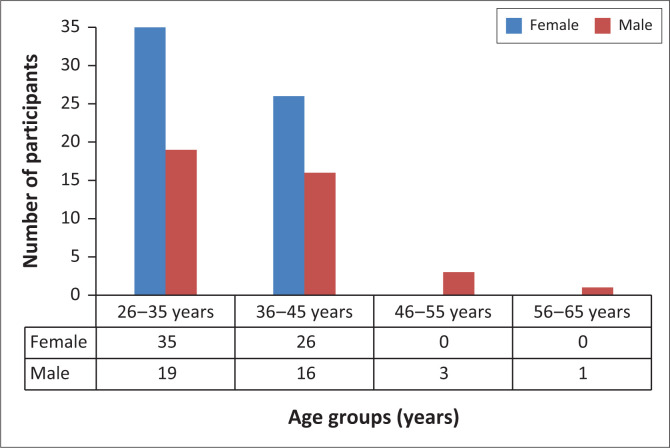
Distribution of survey responders according to age and gender.

A total of 100 participants completed the survey representing a 76% response rate from the proposed 132 participants. Given that the overall acceptability of AI in the sample was 89%, the sample size was deemed sufficient to make inference. The sex distribution of the participants by age classes is presented in Figure 1. The majority of the participants were in the 26–35 years old (54%) age group, and most (61%) were female (Figure 1).

### Knowledge of artificial intelligence

Table 1 shows the participants’ distribution according to their knowledge of AI. All participants indicated that they did not use AI to perform their daily duties. When asked of their ability to work with data, most participants (78%) indicated that they were unable to work with large quantities of data. Similarly, 78% of participants understood the basics of AI; only 3% had no knowledge of AI at all. Sixty per cent believed that they knew the role of AI in radiology while 20% were unsure. Regarding sources of information, 77% indicated that their knowledge about AI was based on what was shared in the media. The majority of participants had never attended either a conference or a course of AI in radiology (93%) and had never been involved in AI development projects (98%). The majority of the participants (95%) believed that AI has the potential to contribute to the preparation of radiographic reports. Regarding limitations, 97% indicated that they understood the limitations of AI.

**TABLE 1:** Distribution of participants according to their knowledge, perceptions, attitudes towards artificial intelligence and its integration into the registrars’ programme.

Question description	Agree (%)	Disagree (%)	Neutral (%)	Total (%)
**Distribution of partic ipants according to their knowledge of artificial intelligence**				
I am able to work with large quantities of data/Reporting everything imaged in my workplace within expected record time	10	78	12	100
I have a basic understanding of AI	78	3	19	100
I know the role of AI in radiology	60	20	20	100
My knowledge about AI is based on the information shared in the media	77	11	12	100
I attend conferences and courses about AI in radiology	5	93	2	100
I am part of research projects on developing applications of AI	0	98	2	100
AI contribute to the preparation of radiographic reports	95	4	1	100
As radiologists/Radiologist Trainee we use AI software on daily basis to perform my duties	0	100	0	100
I have a basic understanding of the limitations of AI	0	97	3	100
**Distribution of participants according to their perceptions of AI**				
I think certain AI techniques can reliably detect pathological conditions	78	11	11	100
I think that AI makes radiology to be easy	10	77	13	100
I think that AI improves the daily work of radiologist	95	1	4	100
I think that AI is going to shift our activity from diagnostic to interventional radiology	0	94	6	100
AI applications would make radiology more exciting for me	82	10	8	100
AI should be used as a support for evaluating radiological images	94	2	4	100
I think AI algorithms can reach a reliable diagnosis without a radiologist	6	89	5	100
I am in favour of AI in my division or practice	95	0	5	100
AI will not replace human radiologists	89	6	5	100
I think AI will change radiologist work dramatically in the next 5 years	0	95	5	100
If AI was used back then it would have discouraged me from specialising in radiology today	13	85	2	100
**Distribution of participants according to their attitudes towards AI**				
I plan to advance my personal AI knowledge to improve my work performance	94	2	4	100
I am willing to learn about AI application	98	0	2	100
I am willing to help in creating AI software for radiologist to do their work	97	0	3	100
If I were to choose my specialty today, I would choose radiology again with AI applications	99	0	1	100
AI will play an important role in healthcare	97	0	3	100
I have an understanding of the basic computational principles of AI	3	92	5	100
**Distribution of participants according to their attitudes towards incorporation of artificial intelligence into the registrar programme**				
I think that AI will change the way that registrars learn positively	97	1	2	100
Do you think that AI can be taught in the registrar programme	96	0	4	100
Learning about the application of AI is as important as medical skills	87	11	2	100
Learning how to use AI is as crucial as imaging physics training Part 1 FCR examination	68	8	24	100
AI should be an important aspect of the registrar programme in the future	98	0	2	100
AI would result in a thorough registrar programme training in the future	84	7	9	100

AI, artificial intelligence; FCR, Fellowship of the College of Diagnostic Radiologists of South Africa.

### Perceptions towards artificial intelligence

Table 1 shows the distribution of perceptions towards AI among trainee and qualified radiologists in the current study. The majority of participants agreed that AI techniques could reliably detect pathological conditions (78%). They disagreed that AI algorithms can independently reach diagnosis for all radiology modalities and pathology spectrum (89%). They believed that for algorithms to be accurately adopted for diagnosis, they need to go through a developmental stage of machine learning that relies on inputs by experts and in this case, through their expert input, radiologists will play a vital role in creating and shaping these AI algorithms. The majority of participants agreed that AI techniques will improve daily work (95%), were exciting (82%), should be used to evaluate radiologic images (94%); 89% were in favour of AI in their practice. Participants were of the view that AI will encourage and /or increase radiology specialisation (95%). Most participants did not think that AI would make radiology easy (77%), shift radiological activities from diagnostic to interventional (95%), could reach a reliable diagnosis without a radiologist (89%), will not replace human radiologists (95%), dramatically change the scope of work for radiologists in the next 5 years (95%), discourage specialisation in radiology had it already have been implemented (85%). Results from the survey showed that 98–100% of participants thought AI would eventually influence the future of all subspecialties of radiology (data not shown).

### Attitudes towards artificial intelligence

The attitude towards AI had majority agreements in willingness to advancing personal AI knowledge to increase work performance (94%), learn about AI application (98%), willingness to help create AI software (97%), choose to practice radiology with AI applications (99%) and that AI would play an important role in healthcare (97%) (Table 1). On another hand, most participants lacked basic understanding of the computational principles of AI (92%); only 3% indicated that they were knowledgeable (Table 1).

### Attitudes towards incorporation of artificial intelligence into the registrar programme

When participants were asked if AI should be incorporated in the registrar programme, majority agreed that AI will positively change how registrars learn (97%) and could be taught in the registrar programme (96%) (Table 1). It was evident from the survey that learning about AI applications is as important as medical skills (87%), is as crucial as the imaging physics training Part 1 Fellowship of the College of Radiologists of South Africa (68%) (Table 1). Participants thought that AI should be an important aspect (98%) of the registrar programme in the future and would result in thorough registrar programme training (84%) (Table 1).

## Discussion

The current study investigated the perceptions and attitudes of qualified and trainee radiologists towards the implementation of AI in radiology, representing the first such study among South African radiologists in any setting. The respondents in this study were diverse across age and gender. The observation that all participants did not use AI to perform their daily duties is indicative that this field is still in its infancy.^15,16^

Generally, participants interviewed were knowledgeable about AI and its potential role in radiology including preparation of radiological reports. About 78% indicated that they could not work with large quantities of data, suggesting a potential skills gap should AI be considered or start to be implemented in practice. Fortunately, research has shown that most radiologists with insufficient previous information on AI show willingness to upskill in this field.^15^ In the current study, an overwhelming majority (93% – 98%) had never attended a conference, lacked educational background and had never been involved in AI development projects. Results are consistent with various other investigations on attitudes and perceptions towards AI in other countries.^8,15,17^ These findings point to imperative education, skills development and support for conversations pertaining to use of AI in radiology. Results from this investigation are not surprising because the current educational structure provides no support for emerging technologies and current advances in medicine. Indeed, this was further supported by the observation that among participants who knew about AI, their knowledge was based on information from social media. Therefore, it is imperative that academic curricula and research platforms are set up in educational institutions to set up a precedent for theoretical background and application demand for AI in the future.^17^ Knowledge deficits can also be corrected through targeted education and training programmes that aim to enhance collaboration between radiologists and AI experts to facilitate successful integration of AI into radiology.

Overall, this investigation showed that participants were mostly positive about the integration of AI in daily radiology practice and perceived it would be an integral part of radiology – enabling reliable and efficient diagnosis of pathological conditions, ultimately improving daily work outcomes. Similar findings have been reported in other studies,^2,4,5,6^ demonstrating an optimistic view for the impact of AI in future radiology. It is interesting to note that participants in this study disagreed that AI would make radiology easy, shift activities of radiologists from diagnostic to interventional radiology or replace human radiologists. Despite fears of jobs and turf losses among other radiologists,^8,9^ it is generally accepted that AI will augment decision making with radiologists and other healthcare practitioners (especially those referring patients for imaging), taking shared responsibility for the outcomes.^5^ Another study demonstrated that while AI could improve automation during diagnosis, it is unlikely that it could reach a reliable diagnosis on its own,^18^ solidifying the continued need for and importance of radiologists. It has been noted that predictions of AI replacing radiologists are far from real in clinical practice; however, radiologists augmenting their practice with AI are expected to replace those who do not.^19,20^ When asked about the influence of AI on radiology subspecialisation, almost all participants thought it would influence all subspecialities questioned. These results are in close association with other studies in which participants thought that breast-imaging, chest-imaging, neuroradiology,^15^ interventional radiology,^21^ ultrasound,^22,23,24^ and antenatal imaging^25^ would be the most impacted by AI. The agreement for AI use among subspecialties emphasises the need for radiologists to embrace AI as a valuable tool that can augment their expertise and enable them to focus more on patient care. The totality of findings from results of the current and other studies demonstrates the potential role of AI in revolutionising radiology and medicine in general.^18^

Regarding attitudes, findings indicate that the majority of participants had a positive attitude towards incorporating AI into their professional development and future work. Specifically, they agreed that advancing their personal knowledge of AI, learning about AI applications and being willing to help create AI software would improve their work performance; findings that are in accordance with those of Yun et al.^17^ However, most participants also disagreed that they currently have a good understanding of the basic computational principles of AI; findings also observed by Abuzaid et al.^26^ Radiologists with current understanding of AI are self-taught. The challenge with self-teaching, however, is that it involves a high degree of variability and relies on information provided by software vendors whose messaging may be biased and exaggerate their products’ capabilities.^26^ This demonstrates an educational gap for more comprehensive education and training to improve healthcare workers’ knowledge of AI, while leveraging their positive attitudes to facilitate the successful integration of AI into various medical domains.

The study found that a significant majority of participants agreed that AI will positively impact the registrar programme. This suggests that AI is viewed as a valuable tool for enhancing the learning experience and training of registrars. Based on the responses, the study highlights how AI can improve the efficiency and accuracy of registrar tasks, such as data analysis and administrative tasks, allowing registrars to focus more on patient care and medical skills which our questions indirectly ask. Incorporating AI into the registrar programme can lead to a more comprehensive and effective training experience, ultimately enhancing the overall quality of registrar services. Additionally, the study underscores the importance of teaching AI applications alongside medical skills to ensure registrars are well-equipped to navigate the evolving healthcare landscape. Similar recommendations for including AI training in medical schools have been given in other studies.^17,18,27^ Proactive training and collaboration between radiologists and AI experts will be crucial to ensure the successful integration of AI into radiology. Through simulation-based medical training, radiology trainees and radiologists will benefit from created AI-powered simulations in real time for skill training.^28^ For ease of access, these may be in a form of stations located in reporting rooms thus integrating AI into daily practice. Various studies have indicated that incorporating AI into registrar training programmes may be of substantial benefit in preparing future radiologists in terms of AI practical skills development. Furthermore, they suggested that structured AI education can significantly enhance the competency of future radiologists.^29^

### Limitations

While the findings were interesting, the sample may not have been representative of South African trainee and qualified radiologists as the sample was from one medical training circuit. The study did not interview the recommended sample size because of staffing constraints, transfer of radiologists to other hospitals and career advancement of trainees having to move out of the circuit. Printing of hard copy questionnaires may have also limited accessibility for potential participants; using a digital platform like Survey Monkey could have augmented reach and will be considered in a future study. The survey and questionnaire were close-ended and can be improved through focus group discussions and open-ended questions. Moreover, participants completed the questionnaire in their own time and space, which may have caused others to not correctly understand the context of some of the questions. Participation in the study was also voluntary and may have skewed the sample size towards participants with an interest in the subject of AI, thereby introducing sampling bias. This study provides a precedent and follow-up country-wide studies should be conducted with a bigger sample size and expanded questionnaire to further understand the intricacies of participants responses.

### Recommendations for future research

While it is important to document the healthcare practitioners’ attitudes and perceptions, future studies should also be conducted among patients, patient families and the general public’s responses towards implementation of AI in radiology, and more broadly in healthcare. Future research must also investigate the key barriers for institutions in integrating AI technologies and how AI in radiology can be implemented on a national scale, both in the current healthcare setting and the impending National Health Insurance in South Africa. Moreover, studies are needed to address the educational gap regarding reservations and ethical concerns surrounding AI in medicine.

## Conclusion

The findings from this study indicated that AI in radiology is still in its infancy and trainee and qualified radiologists lacked basic understanding of AI despite their largely positive attitudes and perceptions. Lack of knowledge for AI was largely attributed to lack of educational and institutional structures for learning this topic. From the survey, it was clear that participants were positive about the prospects of AI education integration in medical school and imaging physics training for the Part 1 Fellowship of the College of Diagnostic Radiologists of South Africa (FCR) examination. The findings from this study demonstrate an unmet educational need of AI for healthcare practitioners and reflect the urgent need for amendments to the curriculum to include AI education. Therefore, designing large data- and AI-focused educational courses, programmes and conferences is recommended both for trainees and qualified radiologists (continuous education). Additionally, there is an opportunity to implement new policies on continuous education in South African radiology. Overall, this study also found that radiologists were willing to be involved in developing AI technologies; thus, partnerships with AI software providers could provide a platform for more hands-on training and could lead to a clear paradigm shift in the understanding and update of AI technologies. The field of AI is growing, and healthcare professionals will also need to evolve with it; thus, the need to leverage current positive attitudes and perceptions.
